# The Waste-to-Biomethane Logistic Problem: A Mathematical Optimization Approach

**DOI:** 10.1021/acssuschemeng.4c01429

**Published:** 2024-05-22

**Authors:** Víctor Blanco, Yolanda Hinojosa, Victor M. Zavala

**Affiliations:** † Institute of Mathematics (IMAG), Universidad de Granada, Granada 18001, Spain; ‡ Dpt. Quant. Methods for Economics & Business, Universidad de Granada, Granada 18071, Spain; ¶ Institute of Mathematics (IMUS), Universidad de Sevilla, Sevilla 41012, Spain; § Dpt. Applied Economics I, Universidad de Sevilla, Sevilla 41018, Spain; ∥ Dpt. Chemical and Biological Engineering, University of Wisconsin-Madison, Madison, Wisconsin 53706, United States; ⊥ Wisconsin Institute for Discovery, University of Wisconsin-Madison, Madison, Wisconsin 53715, United States

**Keywords:** Logistics, Green Energy, Facility Location, Mathematical Optimization, Biogas, Supply Chain

## Abstract

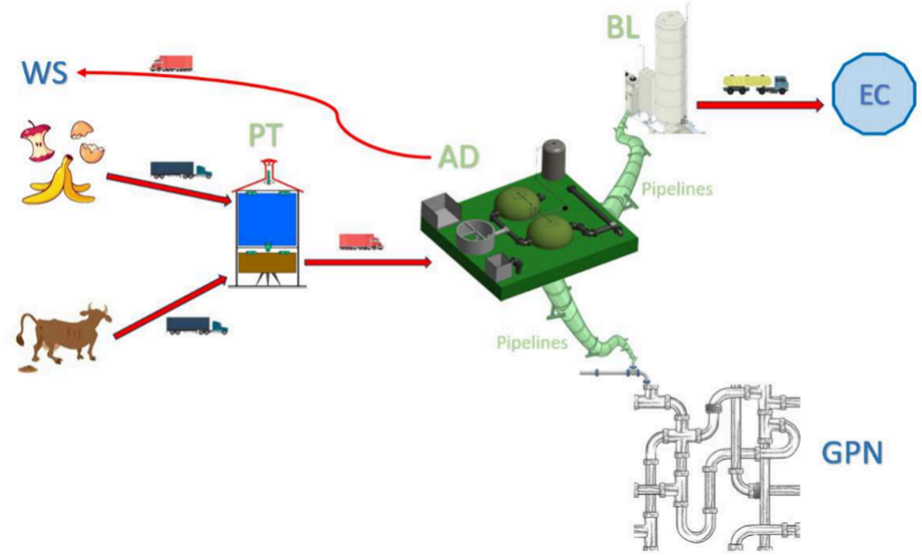

In this paper, we propose a new mathematical optimization approach to make decisions on the optimal design of the complex logistic system required to produce biogas from waste. We provide a novel and flexible decision-aid tool that allows decision makers to optimally determine the locations of different types of plants (pretreatment, anaerobic digestion, and biomethane liquefaction plants) and pipelines involved in the logistic process, according to a given budget, as well as the most efficient distribution of the products (from waste to biomethane) along the supply chain. The method is based on a mathematical optimization model that we further analyze and that, after reducing the number of variables and constraints without affecting the solutions, is able to solve real-size instances in reasonable CPU times. The proposed methodology is designed to be versatile and adaptable to different situations that arise in the transformation of waste to biogas. The results of our computational experiments, both in synthetic and in a case study instance, prove the validity of our proposal in practical applications. Synthetic instances with up to 200 farms and potential locations for pretreatment plants and 100 potential locations for anaerobic digestion and biomethane liquefaction plants were solved, exactly, within <20 min, whereas the larger instances with 500 farms were solved within <2 h. The CPU times required to solve the real-world instance range from 2 min to 6 h, being highly affected by the given budget to install the plants and the percent of biomethane that is required to be injected in the existing gas network.

## Abstract 2

Illustration of the Waste-to-Biomethane Logistic problem, a complex location and distribution problem behind the imminent transition to sustainable energy.

## Introduction

The decarbonization of energy has emerged as a critical global priority in recent years, due to the urgent need to combat climate change. Most world organizations have recognized the significance of transitioning to cleaner and more sustainable energy sources in the next few years to reduce greenhouse gas emissions. Different agreements have been signed to promote this change. Specifically, the United Nations, through its Sustainable Development Goals, has set different targets to ensure affordable, reliable, sustainable, and modern energy by 2030.^1^ The International Energy Agency (IEA) has been actively promoting decarbonization efforts by providing policy recommendations, conducting research, and facilitating international cooperation.^2^ Additionally, the Intergovernmental Panel on Climate Change (IPCC)^3^ has been instrumental in assessing the impacts of climate change and highlighting the importance of decarbonizing the energy sector. In 2019, the European Commission presented the European Green Deal,^4^ the roadmap that Europe should follow for the implementation of the United Nations’ Sustainable Development Agendas for 2030 and 2050, which is designed to mitigate the effects of climate change. Various measures have been taken by these organizations, including advocating for renewable energy investments, promoting energy efficiency, encouraging the use of electric vehicles, and supporting the development of innovative technologies such as carbon capture and storage. These collective efforts by world organizations are crucial in driving the global transition toward a low-carbon future and mitigating the adverse effects of climate change.

One of the challenges is to decarbonize the energy system by developing a new power sector based on renewable sources. One of the main strategies to achieve the proposed goal is the use of biogas as an alternative renewable energy source to carbon-based energies, since it contributes not only to the reduction of greenhouse gases but also to the development of the circular economy through the anaerobic digestion of organic waste from different sources and its transformation to fuel. Since biomethane is the same molecule as natural gas, it can be distributed via the existing gas distribution networks, facilitating the transition from natural gas to biogas energy. Thus, the installation of anaerobic digestion plants for the conversion of organic waste and livestock manure to biomethane has gained prominence in recent years as a sustainable waste-to-energy solution. Many countries are assessing different alternatives to install and adapt these biogas plants. For instance, the U.S. Farm Bill,^5^ periodically reauthorized by Congress, includes provisions that support the development and utilization of biogas from agricultural sources. The bill provides funding for biogas projects, such as the installation of anaerobic digester plants on farms or near them, which capture methane emissions from manure and convert them to usable biogas for electricity generation or transportation fuel.

Analyzing the logistic systems behind the implementation of different modes of energy, particularly biogas, is of paramount importance in ensuring the successful and sustainable integration of renewable energy sources to our global energy landscape. Biogas, derived from organic waste materials through anaerobic digestion, presents a promising alternative to conventional fossil fuels (see refs (6) or (7) for further details on the recent technological breakthroughs in anaerobic digestion of organic biowaste for biogas generation). However, their widespread adoption hinges on addressing intricate logistic challenges. Understanding the logistics involved in the collection, transportation, and processing of organic feedstocks is crucial for optimizing efficiency and minimizing environmental impact. Rigorous analyses on the logistical aspects of biogas production in different countries reveal opportunities for streamlining supply chains, enhancing resource utilization, and reducing overall costs see, e.g., refs (8 and 9). Effective logistic planning also ensures reliable and consistent feedstock availability, which is a key factor in the stable operation of biogas plants. By delving to the logistics of biogas implementation, we can develop strategies that not only bolster the economic viability of this renewable energy source but also contribute to the broader goal of achieving a more sustainable and resilient energy infrastructure.

Among the decisions to be made in the design of an efficient logistic system, the most critical one is the selection of the *best* sites to locate the different types of plants involved in the system (biogas plants, pretreatment plants, and liquefaction plants). This is a difficult task that involves different agents, production and conversion technologies, and types of demand centers. Furthermore, this placement must be coordinated with the different distribution processes.^10^ Mathematical optimization is known to play a very important role in these types of decisions. For instance, mathematical optimization tools have been applied by Tampio et al.^11^ for the design of a cost-optimal processing route for a biogas anaerobic digestion plant to produce fertilizer products based on specified regional needs. Balaman and Selim^12^ developed a mixed integer linear programming model to determine the appropriate locations for the biogas plants and biomass storages. Scarlat et al.^13^ provided a spatial analysis algorithm that uses data of manure production and collection to assess the spatial distribution of the biogas potential in Europe, in order to decide the location of bioenergy plants. A geographic information system-based analysis was used by Valenti et al.^14^ to determine the size and location of four biogas plants in Catania (Sicily, Italy). A multiobjective optimization approach for the optimal location of biogas and biofertilizer plants at the maximum profit and the minimum environmental impact is presented in the work of Díaz-Trujillo and Nápoles-Rivera,^15^ and it is applied for a geographical region in Mexico. Mathematical methods have been used to study the location of bioenergy plants in many other regions and countries (Park et al.^16^ in North Dakota, Sultana and Kumar^17^ in Alberta, Egieya et al.^18^ in Slovenia, Amigun and von Blottnitz^19^ in Africa, Silva et al.^20^ in Portugal,^21^ in Nigeria, Soha et al.^22^ in Hungary, Igliński et al.^23^ in Poland, Delzeit and Kellner^24^ in Germany). Hernandez and Martin^25^ proposed a nonlinear optimization model to compute the optimal operating conditions of the reactors, the biogas composition, and the content of nutrients in the digestate in the production of biodiesel from waste via anaerobic digestion. Hu et al.^26^ gave a mathematical optimization framework to analyze the economical and environmental benefits of recovering biogas, caproic acid, and caprylic acid from different sources of organic waste. Ankathi et al.^27^ considered a mixed integer linear optimization model to determine the location and capacities of biogas plants based only in the location and production of the farms. In terms of locational decisions, some of the classical mathematical approaches have been already adapted to incorporate *green goals* (see, e.g., ref (28)), although more advances are expected within the next few years, based on what our society needs.

On the other hand, mathematical optimization is recognized as a fundamental tool for the design and modeling of multiple logistics, transportation, and supply chain problems (see, e.g., refs (21 and 29−37), among many others). Specifically, mathematical optimization is particularly useful to construct robust and effective supply chains to integrate bioenergy in any economy (see, e.g., refs (38−42) and references therein). In order to implement an efficient and sustainable biogas distribution system, it is necessary to adequately design a robust logistic plan for all the elements involved in the process, such as manure, waste, biomethane, liquefied gas, biofertilizer, etc. In this phase, one must decide not only where to locate the anaerobic digestion plants but also where to locate pretreatment plants for preparing, cleaning, or drying the waste, or transshipment plants to distribute the products, how to collect the manures from the farms or fields and send them to the plants, how to link (if needed) the different types of plants, how to distribute the final biomethane to the gas distribution network or, once it has been previously liquefied, to external clients, how to dispatch possible fertilizers back to some of the farms, etc. A few works have already proposed models to make *optimal* decisions in this type of situations. Jensen et al.^43^ proposed a minimum cost flow-based model for finding the optimal production and investment plan for a biogas supply chain by means of minimizing the transportation cost on an existing supply chain network. Three layers of analysis for designing optimal animal waste supply for anaerobic biodigestion are detailed by Mayerle and Neiva de Figueiredo:^44^ (1) identification of the optimal locations for the anaerobic digestion plants based on the farms’ information; (2) specification of the optimal distribution system; and (3) scheduling the optimal biomass collection from each farm to minimize biogas loss. Sarker et al.^45^ studied the optimization of the supply chain cost for a biomethane gas production system, which is organized in four stages (collecting feedstock to hubs located according to zip code areas, transporting feedstock from hubs to reactor(s), transporting biomethane gas from reactor(s) to condenser(s), and shipping the liquefied biomethane gas from condensers to final demand points). The problem studied by Sarker et al.^45^ is formulated as a mixed-integer nonlinear mathematical optimization problem and, due to its complexity, a genetic heuristic algorithm is proposed to solve it. Abdel-Aal^46^ provided a mathematical optimization model (together with a metaheuristic solution) to maximize the profit of a biomass supply chain designed to commercialize electricity. The model allows us to determine the electricity demand, power plant operations, biomass feedstock purchase and storage, and biomass transport trucks.

In this paper, we introduce the waste-to-biomethane logistic problem and provide a general and flexible mathematical optimization model to make decisions of locating pretreatment, biogas, and liquefaction plants together with the pipelines linking the biogas plants with either the liquefaction plants or the injection points of the existing gas network and distributing the different types of elements along a complex logistic system that integrates all the aspects mentioned above. Thus, we consider a supply chain for the biomethane gas production combining six stages:

(A) collecting waste and sending it to pretreatment plants (regardless of zip code);
(B) delivering contaminant-free organic waste from pretreatment plants to the anaeorobic digestion plants;
(C) constructing pipelines for transporting biomethane from the anaerobic digestion plants to the injection points of an existing gas pipeline network;
(D) constructing pipelines for transporting biomethane from the anaerobic digestion plants to the biomethane liquefaction plants;
(E) dispatching biofertilizers from the anaerobic digestion plants to the waste sources; and
(F) shipping liquefied biomethane from the biomethane liquefaction plants to external customers.

We jointly integrate all of these complex stages to a mixed integer linear programming (MILP) model seeking to minimize the overall transportation cost of the system by assuming that a limited budget is given to install all of the different types of plants and pipelines. Our MILP model can be efficiently solved using off-the-shelf optimization solvers (such as Gurobi, CPLEX, or FICO) after proving some theoretical results for reducing the number of variables and constraints. Then, we first test our method to synthetic (but realistic) instances and show that our proposal is a valid decision making tool for situations where the logistics behind energy transformation is required to be optimized. We also apply our model to a real-world dataset based on the region of upper Yahara Watershed in the state of Wisconsin (see refs (47 and 48) for further information about this dataset).

The remainder of this paper is organized as follows. In the next section, we introduce the Waste-to-Biomethane Logistic Problem (W2BLP) and present our modeling assumptions. The next section is devoted to detailing the mathematical optimization model that we developed for the problem. We also prove in this section a theoretical result that allows us to significantly reduce the number of variables and constraints in the model. After that, we report the results of our computational experiments on realistic synthetic instances. A case study is also presented where the model is applied to the real-world dataset based on the region of the upper Yahara Watershed. The paper concludes with some conclusions and future research.

## The Waste-to-Biomethane Logistic Problem

The problem under analysis consists of efficiently using agricultural waste, such as livestock manure, energy crops, municipal waste, etc., to be transformed to biomethane. This transformation consists of different phases. First, from a given set of waste sources (WS), as farms or residual storages, each of them producing an amount of waste, it is transported to the pretreatment (PT) plants, where the nonorganic material is removed, and what remains is dried, pressed, or adequately prepared. Feedstocks with low dry matter content, such as pig slurry, are often preseparated before digestion to a liquid and a solid fraction, so that only the solid fraction is supplied to the biogas plant. Solid–liquid separation is used to reduce the volumes and the costs of the feedstock transport.^49,50^ Additionally, as already observed by several authors (see, e.g., refs (51 and 52), among others), the proper pretreatment of the biowaste for biogas production reduces environmental pollution and enhances the recovery of renewable energy. In this first phase, the location of the PT plants is determined among a finite set of potential positions.

The dried contaminant-free organic waste (DOW) obtained after the pretreatment is delivered to an anaerobic digestion (AD) plant, where it is transformed to biomethane. The positions of the AD plants are also to be decided among a finite set of potential locations. Biomethane is then either directly injected to the existing gas pipeline network (GPN) or processed to liquid natural gas (LNG). We assume that we are given a finite set of injection points in the GPN and that the pipelines connecting the AD plants with a selected set of injection points are to be built (with a given cost). A minimum percentage of the produced biomethane is to be injected in the GPN, and the remaining biomethane is transformed to liquefied natural gas (LNG) and distributed to a given set of external customers (EC), each of them with a given demand. This minimum percentage represents the tradeoff between the gas injection to the network, with respect to the amount of gas served to the external customers. The liquefaction of biomethane requires the construction of biomethane liquefaction (BL) plants, as well as pipelines connecting the AD plants with them. Then, the liquified gas is delivered to the customers by using tank trucks. Finally, the remaining material in the DOW to biomethane transformation process (digestate) is then returned back from the AD plants to some of the WS, where it can be used as a biofertilizer. In Table 1, we summarize the notation used for the different elements involved in the problem.

**Table 1:** Notation for the Different Elements Involved in the Problem

notation	description
WS	waste sources
PT	pretreatment plants
AD	anaerobic digestion plants
BL	biomethane liquefaction plants
EC	external customers
GPN	gas pipeline network
DOW	dried organic waste
LNG	liquefied natural gas

**Figure 1:**
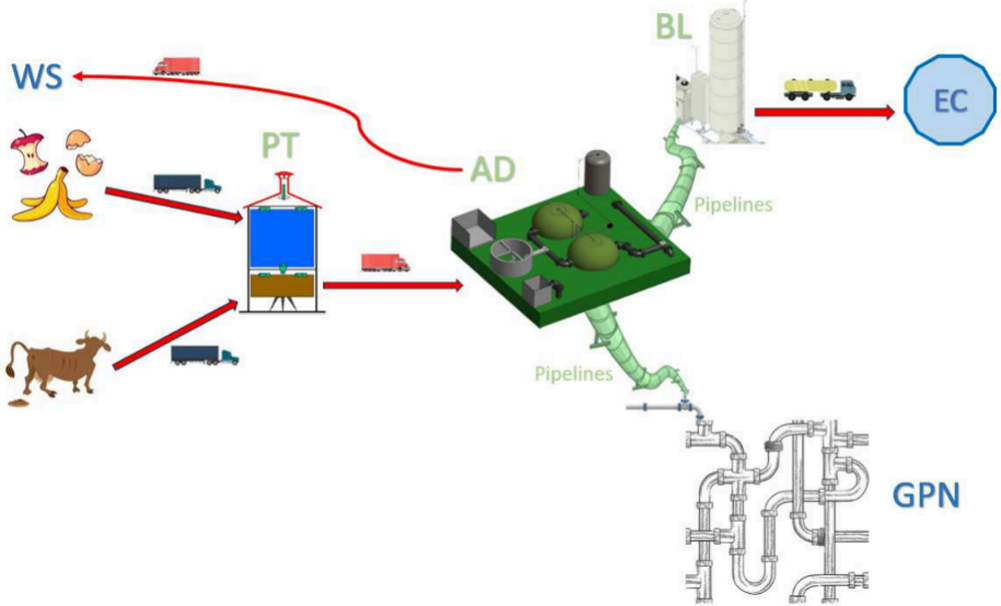
Process modeled in the problem.

In Figure 1, we illustrate the different elements that appear in this logistic problem. The names are differentiated by color. We indicate the input data (WS, GPN, and EC) in blue, whereas we highlight the decisions to be made in green: these are the (PT, AD, and BL) plants and the (to GPN and BL) pipelines to be installed. The transportation routes are highlighted with red arrows in the plot.

In what follows, we summarize the hyphotesis mentioned above that are based on the technological requirements of the process:

- A minimum percentage of the total amount of waste in the WS must be collected and delivered to the PT plants to satisfy the gas requirements of the GPN. Since a given percent of the produced biomethane is to be injected in the GPN, this hypothesis implies that a minimum amount of biogas must be produced to be injected to the network. The aim of this assumption is to ensure that a minimum amount of the production of biogas is supplied to the existing network, but not forcing the collection of all the waste from the WS in case this is not productive for the decision maker.
- Each PT plant sends its whole produced DOW to the AD plants. In the AD plants, the DOW received is transformed to biomethane. This transformation generates an amount of digestate that can be delivered to the WS in the form of biofertilizer.
- We assume that each WS receives a given proportion of the total amount of digestate produced at the AD plants. This assumption allows the WS to get benefit for giving the waste for producing energy, apart from get rid of the waste produced in their lands. In case this proportion is 0, the WS does not receive any biofertilizer (as in the case of residual storages that may not be interested in the product). For this reason, we do not force delivering the whole digestate produced in the AD plants to the WS.
- The whole biomethane production obtained in the AD plants is delivered to the GPN and the BL plants. As already mentioned, a minimum percentage of the total biomethane production is required to be injected to the GPN. The aim of this assumption is to ensure that a minimum amount of the production of biogas is supplied to the existing network. Otherwise, the whole production would be delivered to the external customers, since it implies a positive profit to the distribution company.
- Each BL plant sends its whole LNG production to the EC.
- The demand of the EC is not assumed to be fully satisfied, since the production of biomethane (which depends on the production of waste) might not be enough to satisfy their whole demand. Instead, we consider that every EC receives a prespecified proportion of the total LNG produced at the BL plants. This proportion may depend on its demand or other preference criterion that the agents consider reasonable. This assumption is flexible and allows the decision maker to share the amount of biogas to be delivered to the external customers as desired. A possible choice is to share the liquified biogas proportional to the demand of the customers, but the decision maker may prefer to give priority to some strategic customers.
- A percent of the amount (weight and volume) of the different products is assumed to be lost at each phase of the whole process. Specifically, a given percentage of the amount of waste, DOW, and biomethane received in PT, AD, and BL, respectively, is lost during the process. This technical hyphotesis is derived from the chemical processes to obtain the different types of products from their raw materials.

The efficient management of this system requires both deciding the location of the different types of plants (PT, AD, and BL) and pipelines (connecting AD with GPN and AD with BL) to be constructed, as well as the distribution of the product along the different phases of this process.

There is a cost for opening each potential location of each different type of plants and a construction cost per unit length for the pipelines. We assume that a budget is given to install all of the plants and pipelines.

For the distribution of the products, we assume that a unit transportation cost is provided for the different phases, namely, delivering from WS to the PT plants (waste), from PT to the AD plants (DOW), from the BL plants to the EC (LNG), and from AD to the WS (biofertilizer).

The Waste-to-Biomethane Logistic Problem (W2BLP) consists of deciding the location of the different plants and pipelines and the way the product is distributed, minimizing the overall transportation cost with the given installation budget.

**Figure 2:**
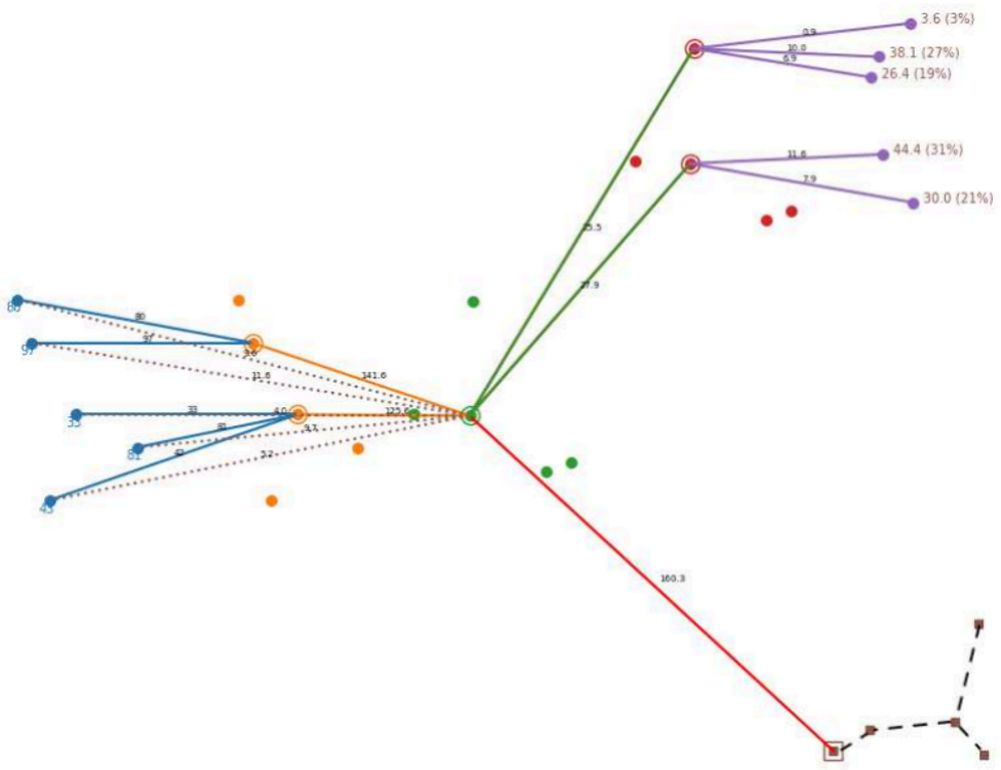
Solution of W2BLP for a toy instance.

In Figure 2, we show a solution of a toy instance of the W2BLP. In such an instance, we consider 5 farms (blue dots in the left side of the plot) with production written at the left of the dot, 5 external customers (purple dots in the right side), and 5 injection points (brown squares in the right side linked with dashed lines in the bottom right side of the plot). Additionally, from left to right, we consider 5 potential locations for the PT plants (orange dots), 5 potential locations for the AD plants (green dots), and 5 potential locations for the BL plants (red dots). Among the potential positions for the plants, the W2BLP decides which of them to open (in this case, those with larger circles), as well as the amount of product that is delivered between the different elements in the system). For instance, the waste produced at the two left top farms (80 and 97 units, respectively) is delivered to the top open PT plant. This plant receives 177 units of waste, and after processing them, 80% (141.6 units of DOW) is delivered to the AD plants. Since only one AD plant is opened, all this production is received in there (together to the rest of the DOW production from the other PT plant). In total, the AD plant receives 266.6 units of DOW. Of this product, after processing it and producing biogas, it is delivered to the injection points and to the EC. The total biogas produced is 80% of the volume of the DOW received from the PT plants (213.28 units). In this solution, 160.3 units are delivered to a single injection point. The rest of the biogas (52.98 units) is delivered to the BL plants. Since two BL plants are opened, one part is delivered to each of them (25.5 and 27.9 units, respectively). Then, the BL plants deliver the LNG (70% of the total amount of biogas received at the BL plants) to the EC. In this case, we assume that, of this production, the LNG is delivered proportionally to the demand of the EC (highlighted at the right of the EC nodes in the plot—in absolute and in percentage in parentheses). The LNG delivered to each of the EC is indicated over the lines linking the BL plants to each of the EC. Finally, the dotted lines represent the biofertilizer delivered from the AD plants to the WS (15% of the volume of the DOW received from the PT plants). We assume that, of this production, the biofertilizer is delivered proportionally to the waste production at the WS. As can be observed, the logistic system is complex and requires making decisions in different layers that affect the others.

## Mathematical Optimization Model

In this section, we present the mathematical optimization model that we propose for the W2BLP. First, we define the parameters and variables that we use in our model.

### Parameters

The following parameters are assumed to be given to our model.

#### Index Sets

We use the following terms to denote the number of elements within each of the index sets: *n* is the number of WS; *m*_1_ is the number of potential locations for the PT plants; *m*_2_ is the number of potential locations for the AD plants; *m*_3_ is the number of potential locations for the BL plants; *d* is the number of EC; and *s* is the number of injection points in the GPN. Then, we use the following notation for the index sets:

- *N* = {1, ..., *n*}: index set for the set WS.
- *P*_1_ = {1, ..., *m*_1_}: index set for the set of potential PT plants.
- *P*_2_ = {1, ..., *m*_2_}: index set for the set of potential AD plants.
- *P*_3_ = {1, ..., *m*_3_}: index set for the set of potential BL plants.
- *I* = {1, ..., *s*}: index set for injection points in GPN.
- *C* = {1, ..., *d*}: index set for the set of EC.

#### Production Parameters

The following production parameters are used in this work:

- *w*_*i*_: waste produced at the *i*th WS, for all *i* ∈ *N*.
- *W* = ∑_*i*∈ *N*_*w*_*i*_: overall production of waste in all of the WS.
- δ: proportion of waste transformed to DOW at the PT plants (0 < δ < 1).
- γ_1_: proportion of DOW transformed to biomethane at the AD plants (0 < γ_1_ < 1).
- γ_2_: proportion of DOW transformed to biofertilizer at the AD plants (0 < γ_2_ < 1). We assume that γ_1_ + γ_2_ < 1.
- β: proportion of biomethane transformed to LNG at the BL plants (0 < β < 1).
- *D*_*l*_: demand of EC*l* ∈ *C*.
- *p*: lower bound percentage of the total waste produced at the WS that must be collected and sent to the PT plants (0 < *p* ≤ 1).
- *q*: lower bound for the total proportion of biomethane produced at the AD plants that must be injected to the GPN (0 < *q* ≤ 1).
- *R*_*i*_: proportion of the total amount of biofertilizer to be delivered to WS *i* ∈ *N* (∑_*i*∈*N*_*R*_*i*_ ≤ 1).
- α_*l*_: proportion of the total amount of LNG at the BL plants that will be received by demand point *l* ∈ *C* (∑_*l*∈*C*_ α_*l*_ = 1).

#### Set-up Costs and Budget

These costs represent the installation costs of the different types of plants and links. They may include not only the construction cost of the different plants and pipelines but also other costs related with the maintenance and use of them, such as labor costs, land costs, etc.:

- *f*_*j*_^1^: set-up cost for opening PT plant *j* ∈ *P*_1_.
- *f*_*j*_^2^: set-up cost for opening AD plant *j* ∈ *P*_2_.
- *f*_*j*_^3^: set-up cost for opening BL plant *j* ∈ *P*_3_.
- *h*_*jk*_: set-up cost for installing a pipeline linking AD plant *j* ∈ *P*_2_ with BL plant *k* ∈ *P*_3_.
- *g*_*jl*_: set-up cost for installing a pipeline linking AD plant *j* ∈ *P*_2_ with the injection point in GPN *l* ∈ *I*.
- B: total budget for installing plants and pipelines.

#### Transportation Costs

These costs represent the transportation costs of the different products (waste, DOW, LNG, and digester solid) that are usually transported by trucks from different geographical locations of the elements in the system:

- *c*_*ij*_^1^ ≥ 0: unit transportation cost of waste from WS *i* ∈ *N* to PT plant *j* ∈ *P*_1_.
- *c*_*jk*_^2^ ≥ 0: unit transportation cost of DOW from the PT plant *j* ∈ *P*_1_ to AD plant *k* ∈ *P*_2_.
- *c*_*jl*_^*C*^ ≤ 0: unit transportation cost (profit) of LNG from the BL plant *j* ∈ *P*_3_ to EC *l* ∈ *C*. Note that this nonpositive cost represents the benefit of delivering to the EC each unit of liquified gas for their particular use.
- *c*_*ji*_^*N*^ ≥ 0: unit transportation cost of biofertilizer from the AD plant *j* ∈ *P*_2_ to WS *i* ∈ *N*.

Note that the transportation costs for the biogas through the pipelines are already included in the setup costs described above.

#### Variables

Our model makes a decision on the different plants and pipelines that are opened as well as the amount of product delivered at the different phases of the process.

##### Binary Variables

The following variables determine whether a plant or a pipeline should be installed:
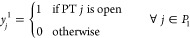

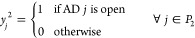

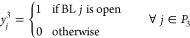

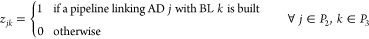

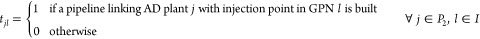


##### Continuous Variables

The following variables of our model decide the amount of product delivered between the different sites in the system:

- *x*_*ij*_^1^: amount of waste delivered from WS *i* to the PT plant *j*, ∀*i* ∈ *N*, *j* ∈ *P*_1_.
- *x*_*jk*_^2^: amount of DOW delivered from PT plant *j* to AD plant *k*, ∀*j* ∈ *P*_1_, *k* ∈ *P*_2_.
- *x*_*jk*_^3^: amount of biomethane delivered from AD plant *j* to BL plant *k*, ∀*j* ∈ *P*_2_, *k* ∈ *P*_3_.
- *x*_*jl*_^*I*^: amount of biomethane delivered from AD plant *j* to GPN’s injection point *l*, ∀*j* ∈ *P*_2_, *l* ∈ *I*.
- *x*_*ji*_^*N*^: amount of digester solid delivered from AD *j* to WS *i*, ∀*j* ∈ *P*_2_, *i* ∈ *N*.
- *x*_*jl*_^*C*^: amount of LGN delivered from BL plant *j* to EC *l*, ∀*j* ∈ *P*_3_, and *l* ∈ *C*.

#### Objective Function

The goal of W2BLP is to minimize the total transportation cost of the system. For that, one must decide the optimal position of the different plants and pipelines with the given budget *B*. These locations have a direct impact on the transportation cost.

With the above set of variables, the overall transportation cost of the system can be written as follows:
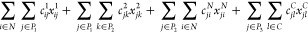
1

#### Constraints

The assumptions of our problem are adequately established by the following constraints:

• The budget for installing the different plants and pipelines must be satisfied:
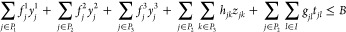
2

• *Flow Conservation Constraints*: The product must be adequately routed through the intermediate stages of the supply chain and enforced by the following constraints:
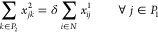
3
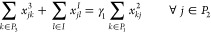
4
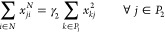
5
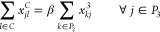
6Constraints (3) ensure that all the waste received at a PT plant is delivered to the AD plants once it is processed (100δ% of the waste is transformed to DOW). 100γ_1_% of the amount of DOW received at each AD plant is transformed to biomethane and fully delivered either to the injection points or to the BL plants (Constraint (4)) and 100γ_2_% is converted to digester solid and delivered to the WS (Constraint (5)). Finally, Constraints (6) ensure that the amount of biomethane received at the BL plants is converted to 100β% of LNG and fully delivered to the EC.

• *Demand and Capacity Constraints:* The different products obtained in the process are adequately delivered according to demand and capacity. Specifically, the WS must send waste and receive digester solid, the EC must receive LNG, and the GPN must receive biomethane. To this end, the following constraints are incorporated to our model:
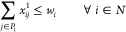
7
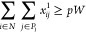
8
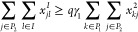
9
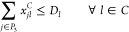
10

Constraints (7) ensure that the amount delivered from each WS is, at most, the waste that it produces. Constraint (8) forces the sending, from the WS’s, of at least 100*p*% of the total amount of waste. Constraint (9) states that at least 100*q*% of the produced biomethane must be injected in the GPN. Each EC, by Constraints (10), at the most, receive its required demand. Note that the transportation cost for the product in this phase is nonpositive, and then, if possible, this demand will be satisfied. Also note that, by using Constraints (3), Constraints (8) and (9) can be equivalently rewritten as
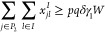
11It may happen that if *q* is large, the overall demand for the EC that can be satisfied is small. Thus, one can establish a sharing rule for the available LNG, i.e., a vector (α_1_, ..., α_|*C*|_) ∈ [0, 1]^|*C*|^ representing the percent of the LNG at the BL plants that will be received by each EC. The following constraints ensure the adequate verification of this share:
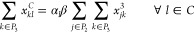
12Finally, Constraints (13) force each WS to receive its given percentage of the total production of digester solid:
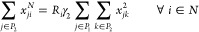
13

• *Distribution through Open Plants and Links*: The transportation of the product in the different phases forces the plants and/or pipelines to be installed. This is ensured by the following constraints:
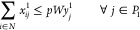
14
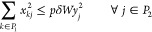
15
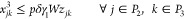
16
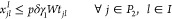
17These constraints ensure that, in case a plant or a link is not open, the product cannot be distributed through the corresponding plant or link.

• *Compatibility of Open Plants and Links*: The installation of a pipeline linking a plant with other plant, or with an injection point in the GPN, is subject to the previous installation of the plants that the pipelines are connecting. These conditions are imposed in our model with the following linear constraints:
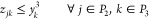
18
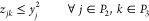
19
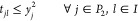
20

Summarizing all the previous descriptions, the W2BLP can be modeled with the following mathematical optimization problem that we denote by (*W2BLP*)_0_:
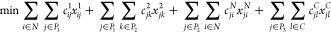
subject to Constraints (2)–(7),Constraints (10)–(20),
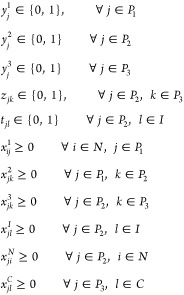
The above model is a mixed-integer linear programming (MILP) problem with *m*_1_ + *m*_2_ + *m*_3_ + *m*_2_*m*_3_ + *m*_2_*s* binary variables, *nm*_1_ + *m*_1_*m*_2_ + *m*_2_*m*_3_ + *m*_2_*s* + *m*_2_*n* + *m*_3_*d* continuous variables and 2*m*_1_ + 3*m*_2_ + *m*_3_ + 3*m*_2_*m*_3_ + 2*m*_2_*s* + 2(*n* + *d*) + 2 constraints. Although commercial solvers are able to handle this type of problem, it can be challenging to solve for real-world instances. Thus, in what follows, we provide a result that allows us to reduce the number of variables and constraints considerably, thus reducing the size of the mathematical optimization model above without affecting the solutions of the W2BLP. Specifically, the result is based on the fact that once the potential AD plants that are open are decided, each of them will optimally distribute the biogas to the GPN through its less costly injection point.

**Lemma 0.1***Let S̅* = (*y̅*^1^, *y̅*^2^, *y̅*^3^, *z̅*, *t̅*, *x̅*^1^, *x̅*^2^, *x̅*^3^, *x̅*^*I*^, *x̅*^*N*^, *x̅*^*C*^) *be an optimal solution of the model* (*W2BLP*)_0_. *If t̅*_*jl*_*= 1 for some j ∈ P* and *l ∈ I, then there exists an optimal solution Ŝ = (y̅*^1^, *y̅*^2^, *y̅*^3^, *z̅*, *t̂*, *x̅*^1^, *x̅*^2^, *x̅*^3^, *x̂*^*I*^, *x̅*^*N*^, *x̅*^*C*^) *with*:
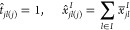

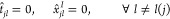
*where l(j) = arg min*_*l∈ I*_*g*_*jl*_*.*

*Furthermore, the overall setup costs of Ŝ is smaller or equal than the ones for S̅.*

*Proof.* The proof that follows is straightforward, since there are no transportation costs between the AD plants and the injection points, but linking them is taken to account in the budget constraint. Thus, replacing the link (*j*, *l*) by (*j*, *l*(*j*)) does not affect the objective function but reduces the setup costs.


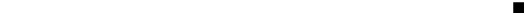


The result above allows us to simplify the model by reducing the number of variables modeling the links and the flows between the AD plants and the GPN. Specifically, one can replace the two-index variables *t* and *x*^*I*^ by one-index variables that, abusing the notation, we denote as
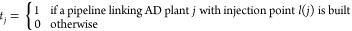
for all *j* ∈ *P*_2_, and *x*_*j*_^*I*(*j*)^ is the amount of biomethane delivered from AD plant *j* to GPN’s injection point *l*(*j*), ∀*j* ∈ *P*_2_.

Thus, if we denote by *g*_*j*_ = *g*_*jl*(*j*)_ for all *j* ∈ *P*_2_, one can replace the two-indices *t* and *x*^*I*^variables in model (*W2BLP*)_0_ by the one-index *t* and *x*^*I*^variables above. Consequently, Constraints (2), (4), (11), (17), and (20) are replaced by the following inequalities and equations:
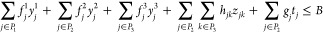
21
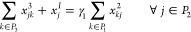
22
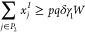
23
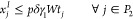
24
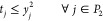
25

The simplified model above has *m*_1_ + 2*m*_2_ + *m*_3_ + *m*_2_*m*_3_ binary variables, *nm*_1_ + *m*_1_*m*_2_ + *m*_2_*m*_3_ + *m*_2_ + *m*_2_*n* + *m*_3_*d* continuous variables, and 2 + 2*m*_1_ + 4*m*_2_ + *m*_3_ + 2*n* + 2*d* + *m*_2_*s* + 3*m*_2_*m*_3_ linear constraints and, as one can observe in the next section, this reduced model is able to solve real instances of the W2BLP in reasonable CPU time. Our approach is then a useful tool for making decisions on the logistic of the biogas production system. Note that in case any of the phases in the process are not present (PT-AD, AD-BL-EC, AD-GPN, or AD-WS), the model above can be simplified, reducing its number of variables and constraints.

## Computational Experiments

We have run a series of experiments to analyze the computational performance of our approach. The main goal of the experiments is to determine the computational limitations of our model and its ability to obtain solutions for real-world instances.

We randomly generated different instances with different sizes and parameters. We generate the coordinates for the WS, the potential location for the different plants (PT, AD, and BL), the injection points in GPN, and the EC uniformly in [0, 1000] × [0, 1000]. For the sake of simplification, we assume that the number and the location of WS (*n*) is the same that the number and the location of potential PT plants (*m*_1_), and that the number and the location of potential AD plants (*m*_2_) and potential BL plants (*m*_3_) are also the same. Additionally, the number of EC atoms (|*C*|) also coincides with the number of injection points in GPN (*s*). The value of *n* = *m*_1_ ranges in {25, 50, 100, 200, 500}, *m* ≔ *m*_2_ = *m*_3_ ranges in {5, 10, 20, 50, 100} with *m* ∈ , and *d* = *s* ranges in {10, 20, 50, 100} with *d* ≤  (here, ⌈·⌉ and ⌊·⌋ stand for the ceiling and floor integer rounding functions, respectively).

Let dist_*ij*_ denote the Euclidean distance between the locations *i* and *j*. We considered as unit transportation costs the Euclidean distances between the different points (dist_*ij*_), except for the transportation costs of LNG from BL plants to the EC, where the unit transportation cost is considered as a profit and is defined as
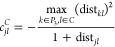
26These costs are designed to represent the concept that the closer the extra customer is to the open BL plant, the larger the profit to satisfy its unit demand.

The setup costs for the PT plants and the BL plants were chosen all equal to one unit (in millions of U.S. dollars) and the installation cost for the AD plants was fixed to 5 units (in millions of U.S. dollars). The setup costs for the pipelines linking the AD plants with the injection points in GPN and the BL plants are 0.1 times the normalized (by the maximum) Euclidean distance between the corresponding points.

For determining adequate budgets for a given instance, we first solved the problem without the budget constraint (Constraint 2). After solving the problem, we compute its effective set-up cost by adding up the costs of the open plants and pipelines that route a positive flow. We use *B̂* to denote this set-up cost and consider as budget *B* = 0.2 *B̂*. This budget indicates that one can use only 20% of the cost of making the best (minimum transportation cost) decision with no budget.

The slurry production at each farm (*w*_*i*_ for *i* ∈ *N*) has been uniformly generated in [1, 100] ∩ _+_ (here,  stands for the set of non-negative integer numbers). The percentage of the overall produced biomethane that must be injected to the existing GPN (*q*) ranges in {50%, 70%, 90%}. We have considered parameters *p* = 1, δ = 0.8, γ_1_ = 0.8, γ_2_ = 0.15, β = 0.7, α_*l*_ =  for all *l* ∈ *C*, and *R_i_* =  for all *i* ∈ *N*. The LNG demand of each EC (*D*_*l*_ for *l* ∈ *C*) was randomly generated in [δγ^1^β, 100δγ^1^β].

The values of the parameters that we consider in our experiments are summarized in Table 2.

**Table 2:** Summary of the Parameters Used in Our Experiments

coordinates	unif[0, 1000]^2^
*n* = *m*_1_	{25, 50, 100, 200, 500}
*m* ≔ *m*_2_ = *m*_3_	{5, 10, 20, 50, 100} with
*d* = *s*	{10, 20, 50, 100} with *d* ≤
*c*_*ij*_^1^, *c*_*ij*_^2^, *c*_*ij*_^*N*^	dist_*ij*_
*c*_*jl*_^*C*^	
*f*^2^	5
*f*^1^, *f*^3^	1
*h*_*jk*_	
*g*_*j*_	
*B*	0.2 *B̂*
*w*_*i*_	Unif[1, 100] ∩
*q*	{50%, 70%, 90%}
δ = γ_1_	0.8
γ_2_	0.15
β	0.7
*p*	1
α_*l*_	
*R*_*i*_	
*D*_*l*_	Unif[δγ^1^β, 100δγ^1^β]

The model has been coded in Python 3.7, using an iMac computer with a 3.3 GHz processor and an Intel Core i7 with 4 cores and 16 GB 1867 MHz DDR3 RAM. We used Gurobi 9.1.2 as the optimization solver. A time limit of 6 h was fixed for all the instances. All the instances with *n* ≤ 100 were optimally solved. For the larger instances, we fixed a MIPGap limit (the solver stops when reaching such a limit and outputs the best feasible solution). For *n* = 200, we fix a MIPGap limit of 2%, and for *n* = 500, an MIPGap limit of 4% is used. For each combination of parameters *n*, *m*, *d* = *s*, and *q*, we solved five instances. Thus, we have solved a total of 450 instances.

**Figure 3:**
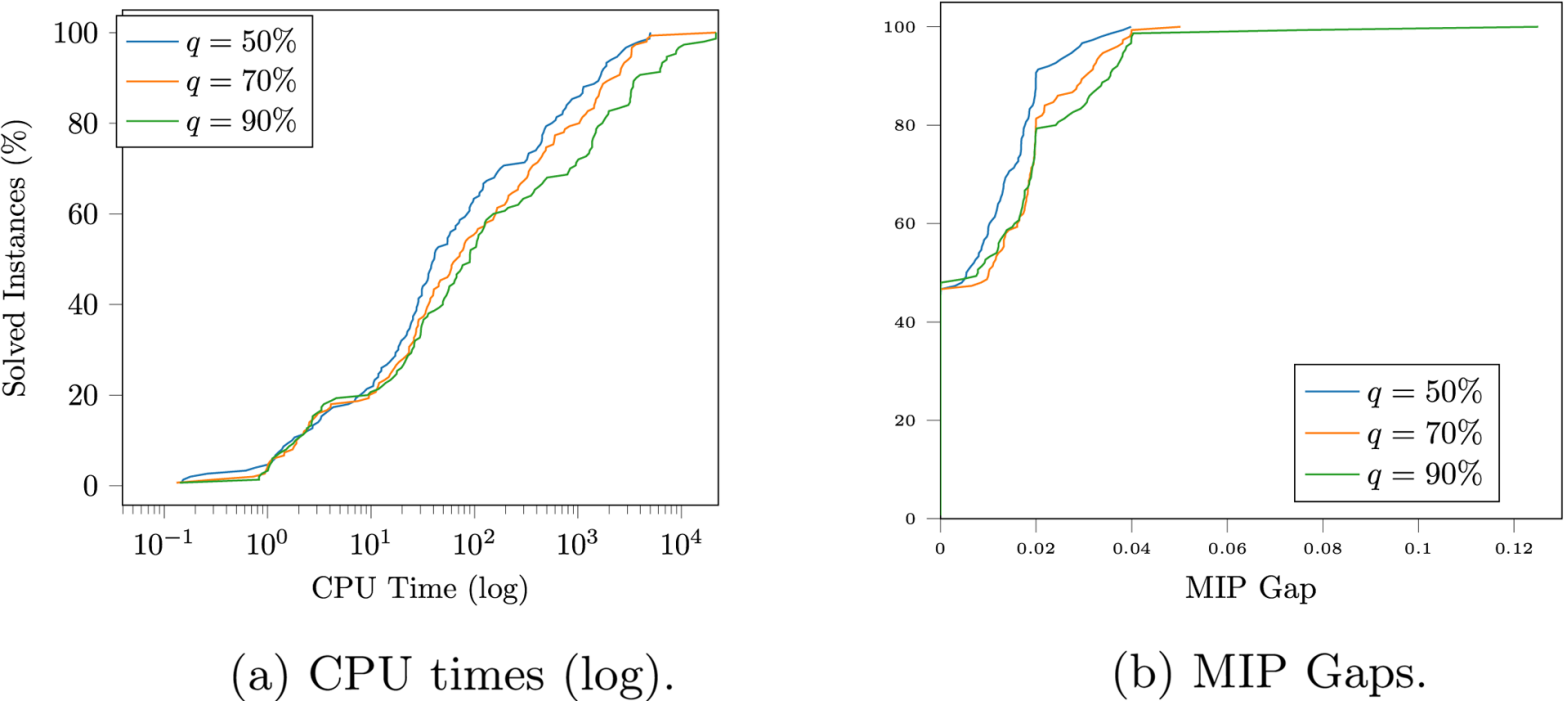
Performance profile ((a) CPU time and (b) MIPGap) of our computational experience for the different values of parameter *q*.

Figure 3 depicts performance profiles, with respect to CPU times and MIP Gaps. In these pictures, we plot the percentage of instances solved up to each value of the CPU or MIP Gap, respectively. We analyze the computational performance of the model for the different values of the parameter *q*. As one can observe, the model seems to perform slightly better for the smaller values of *q*, although the differences are tiny. For this reason, in what follows, we will not differentiate between the different values of *q*.

In Table 3, we summarize the results of our computational experiments. The first three columns indicate the sizes of the instances. Each of the rows summarize the results of a total of 15 instances (5 random instances and 3 values of *q*). Information given in the column with the header “CPUTime” is the average CPU time (in seconds) that the solver required to solve the instances. Information given in the column with the header “MIPGap” indicates the average percent MIP Gap. In the case where the instance is optimally solved, the MIPGap is 0%. Otherwise, this number reports either the MIPGap obtained within the time limit (if it is greater than the MIP Gap limit) or the MIPGap when the MIPGap limit was reached (which can be slightly smaller than the MIPGap limit). Finally, information given in the column with the header “%Unsolved” indicates the percentage instances summarized in the row that were not optimally solved or that reached the MIPGap limit within the time limit.

**Table 3:** Summary of Our Computational Experience

*n*	*m*	*d*	CPUTime	MIPGap	%Unsolved
**25**	**5**	10	0.67	0%	0%
	**10**	10	1.71	0%	0%
**25 total**			**1.19**	**0%**	**0%**
					
**50**	**5**	10	1.58	0%	0%
		20	2.24	0%	0%
	**10**	10	4.55	0%	0%
		20	4.70	0%	0%
	**20**	10	24.78	0%	0%
		20	28.83	0%	0%
**50 total**			**11.11**	**0%**	**0%**
					
**100**	**10**	10	32.08	0%	0%
		20	48.22	0%	0%
		50	28.14	0%	0%
	**20**	10	109.59	0%	0%
		20	181.59	0%	0%
		50	179.04	0%	0%
**100 total**			**96.44**	**0%**	**0%**
					
**200**	**20**	10	444.32	1.47%	0%
		20	137.37	1.69%	0%
		50	70.65	1.44%	0%
		100	51.63	1.40%	0%
	**50**	10	1195.32	1.77%	0%
		20	413.42	1.81%	0%
		50	46.63	1.54%	0%
		100	40.78	1.45%	0%
**200 total**			**300.01**	**1.57%**	**0%**
					
**500**	**50**	10	6946.33	4.45%	20.00%
		20	1041.76	2.90%	0%
		50	912.06	1.93%	0%
		100	713.42	1.74%	0%
	**100**	10	5656.14	3.10%	6.67%
		20	3625.32	2.50%	0%
		50	2366.02	2.47%	0%
		100	3195.16	1.32%	0%
**500 total**			**3057.03**	**2.55%**	**3.33%**
					
**total**			**916.80**	**1.10%**	**0.89%**

As mentioned previously, we observe that all of the instances with up to *n* = 100 have been optimally solved within the time limit. As expected, the computing time increases with the values of *n* and *m*, while it does not seem to depend on the value of *d*. The average computing time over all these instances is ∼46.26 s, being 1.19 s for the instances with *n* = 25, 11.11 s for *n* = 50, and 96.44 for *n* = 100. The average computing time needed to obtain an optimal solution is dependent on the value of *m*: 1.5 s for *m* = 5, 19.9 s for *m* = 10, and 104.8 s for *m* = 20.

Regarding the instances with *n* = 200, we observe that all the instances have been solved, which, in this case, means that all the instances have reached the MIP gap limit within the time limit, being the average consuming time over all the instances 300 s and the average MIP gap 1.57%. One can observe that, for these instances, the computing time is greater than the value of *d* (EC = number of injection points). This may be because the value of *d* does not affect the number of binary variables in our improved formulation, and it might happen that the smaller the number of EC and injection points, the more difficult it is to decide where to install the different plants to satisfy them at optimal cost. This fact can also be observed for the largest instances with *n* = 500. For *d* = 10, 20% of the instances with *m* = 50 and 6.67% of the instances with *m* = 100 have not been solved within the time limit; that is, these instances have not reached the MIP gap of 4% within 6 h. The rest of the instances have been solved within the time limit. The average computing time for all the instances with *n* = 500 was 3057 s, and the average MIP gap was 2.55%.

The results shown in Table 3, together with Figure 3, allow us to get a clear empirical evidence that, using our improved formulation, the problem can be solved (with the MIP gap limit) within the time limit using a commercial solver. In fact, only 4 of the 450 instances (all of them with *n* = 500) have reached the time limit with an MIPGap greater than the MIP gap limit. This number represents <1% of the total instances and only 3.3% of the instances with *n* = 500.

From our experiments, we conclude that our approach provides a useful tool to make decisions about the complex logistic problem behind the energy transformation of waste to biogas in realistic instances using reasonable computational resources. Thus, our model can also be used to evaluate different alternatives, based on different values for the setup costs for the plants or different transportation costs.

## Case Study

**Figure 4:**
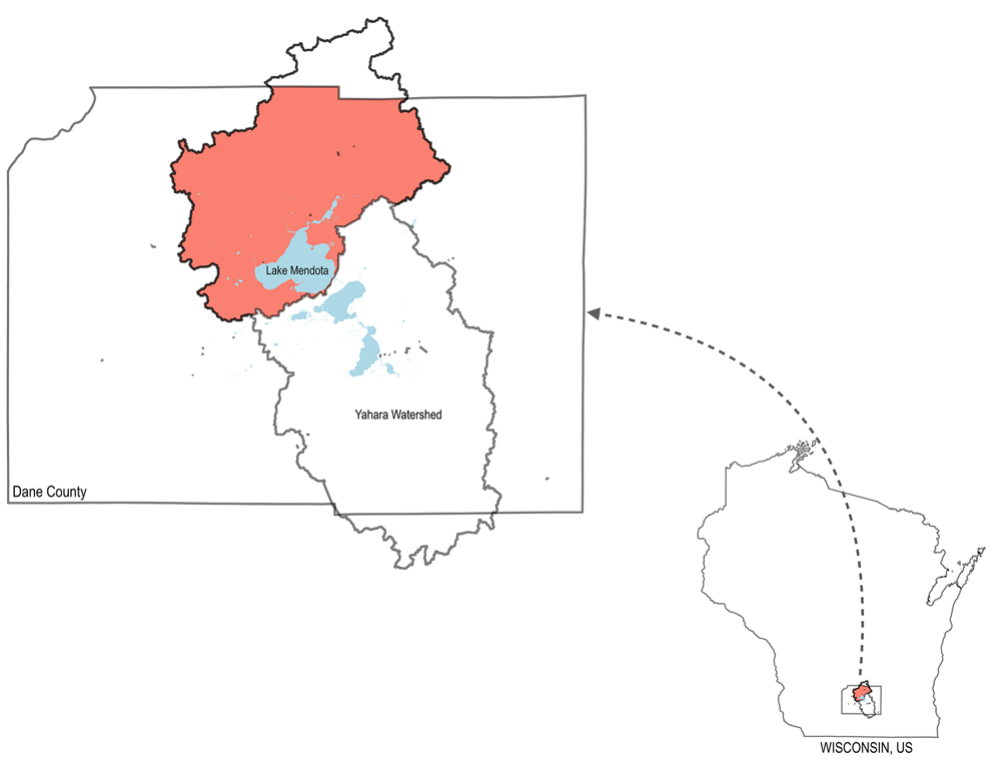
Lake Mendota in the Upper Yahara watershed region in Dane County, WI.

We tested our model in a real-world dataset based on the upper Yahara watershed region in the state of Wisconsin (see Figure 4), the data for which are available at https://github.com/zavalab/JuliaBox/tree/master/Graph_S%26C. A detailed description of this dataset can be found in refs (40, 47, 48, and 53). This region consists of 203 dairy farms whose waste production is known and whose locations are predefined. These locations determine our set of WS and the potential positions for the PT plants. The potential positions for AD plants and BL were randomly generated in that region. The positions of the injection points on the GPN were also randomly generated in this region, and a network connecting these points was constructed. The position of the EC was randomly generated outside that region but in the minimum rectangular box containing it.

**Figure 5:**
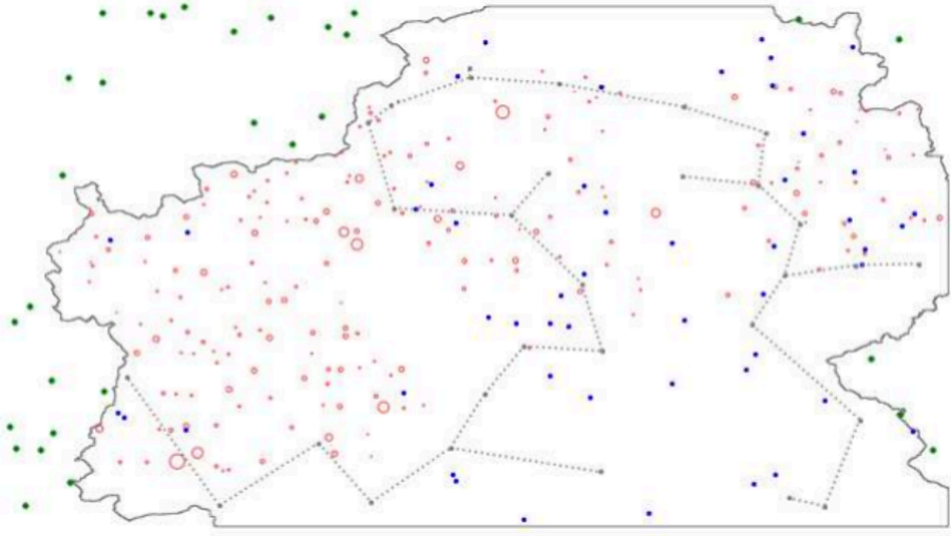
Input data of the case study.

In Figure 5, we plot the coordinates of the different agents involved in the W2BLP. Red points in the plot indicate the WS locations (as larger the size of the dot, larger the production of waste in the farm) and the potential locations for PT, blue points are the potential locations for AD plants and for BL plants, green points indicate the EC locations, gray dotted lines represent the GPN, and gray points denote the injection points of the GPN.

The parameters not provided in the above repository were fixed as follows:

- *c*_*ij*_^1^ = 0.3 × dist_*ij*_, for *i* ∈ *N* and *j* ∈ *P*_1_
- *c*_*jk*_^2^ = 0.15 × dist_*jk*_, for *j* ∈ *P*_1_ and *k* ∈ *P*_2_
- *c*_*ji*_^*N*^ = 0.15 × dist_*ji*_, for *j* ∈ *P*_2_ and *i* ∈ *N*
- *D*_*l*_ was randomly generated in [δγ_1_β × min *w*_*i*_, δγ_1_β × max *w*_*i*_]
- The setup budget was fix to κ × *B̂*, for κ ∈ {0.1, 0.2}, where *B̂* was computed as described in the previous section:
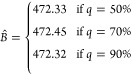
- The rest of parameters have been considered as described in the previous section (*q* ranges in {50%, 70%, 90%}, *p* = 1, δ = 0.8, γ_1_ = 0.8, γ_2_ = 0.15, β = 0.7, α_*l*_ =  for all *l* ∈ *C*, and *R_i_* =  for all *i* ∈ *N*).

The parameters required to reproduce the obtained results are available at https://github.com/vblancoOR/w2blp.

As in the previous section, we set a time limit of 6 h for solving the problem. In Table 4, we show the CPU times (in seconds) and MIP Gaps obtained after running our model in the six different configurations of budget and *q* that we consider. In that table, one can observe that, except for the case *B* = 0.1*B̂* with *q* = 0.9, all of the instances were optimally solved within the time limit. The case were the optimality is not guaranteed, we obtained a MIP gap of 0.1% which is negligible. Furthermore, as expected, a more-limited budget has a significant impact in the CPU time required to solve the problem, the problems with budget 0.1*B̂* being more challenging than those with budget 0.2*B̂*.

**Table 4:** CPU Times and MIP Gaps for Solving the Instances in the Case Study

*B*	*q*	CPU Time (s)	MIPGap
0.1 *B̂*	0.5	2595.82	0%
	0.7	18093.03	0%
	0.9	21602.8	0.1%
			
0.2 *B̂*	0.5	84.67	0%
	0.7	473.81	0%
	0.9	3106.91	0%

**Figure 6:**
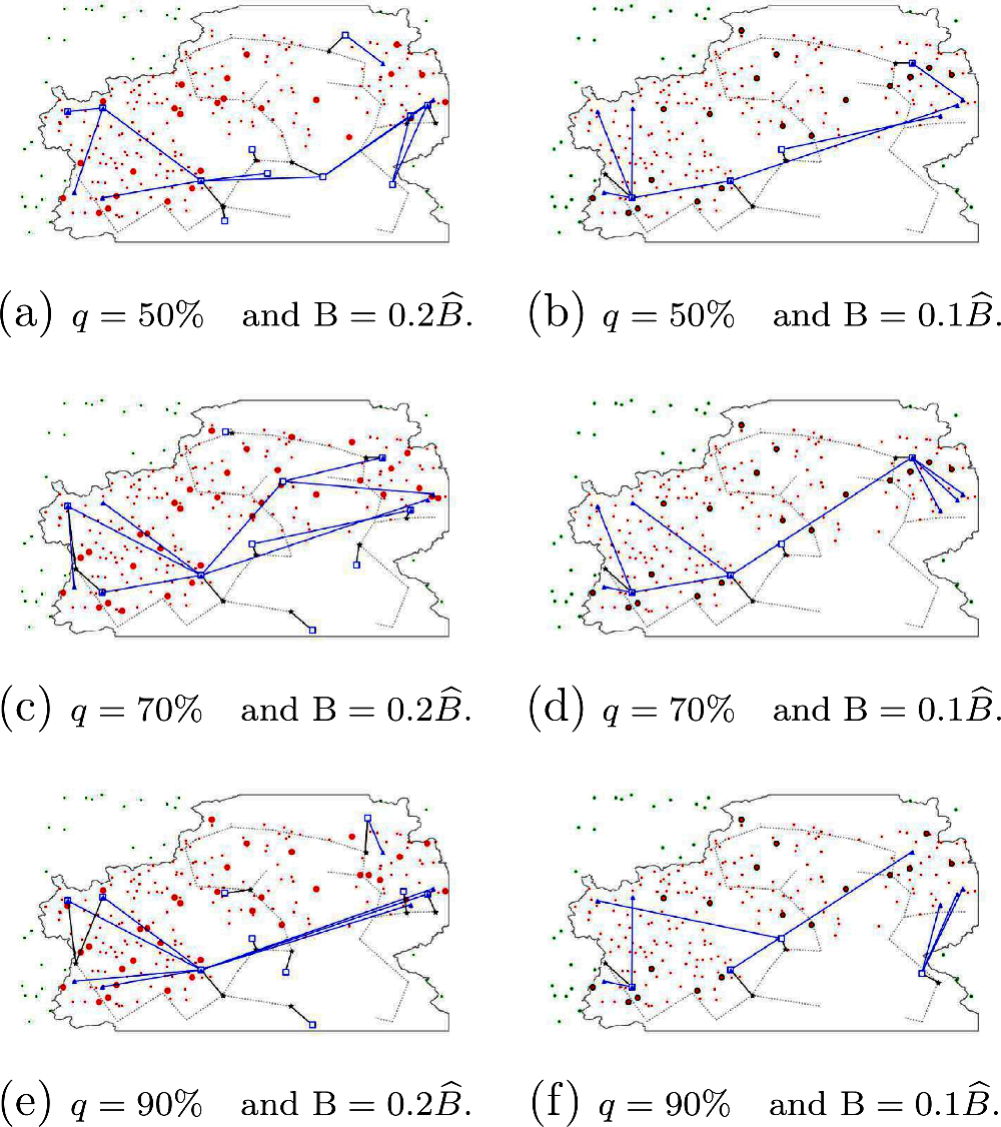
Optimal location of the different types of plants and pipelines for different values of *B* and *q*: (a) *q* = 50% and *B* = 0.2*B̂*, (b) *q* = 50% and *B* = 0.1*B̂*, (c) *q* = 70% and *B* = 0.2*B̂*, (d) *q* = 70% and *B* = 0.1*B̂*, (e) *q* = 90% and *B* = 0.2*B̂*, and (f) *q* = 90% and *B* = 0.1*B̂*.

**Figure 7:**
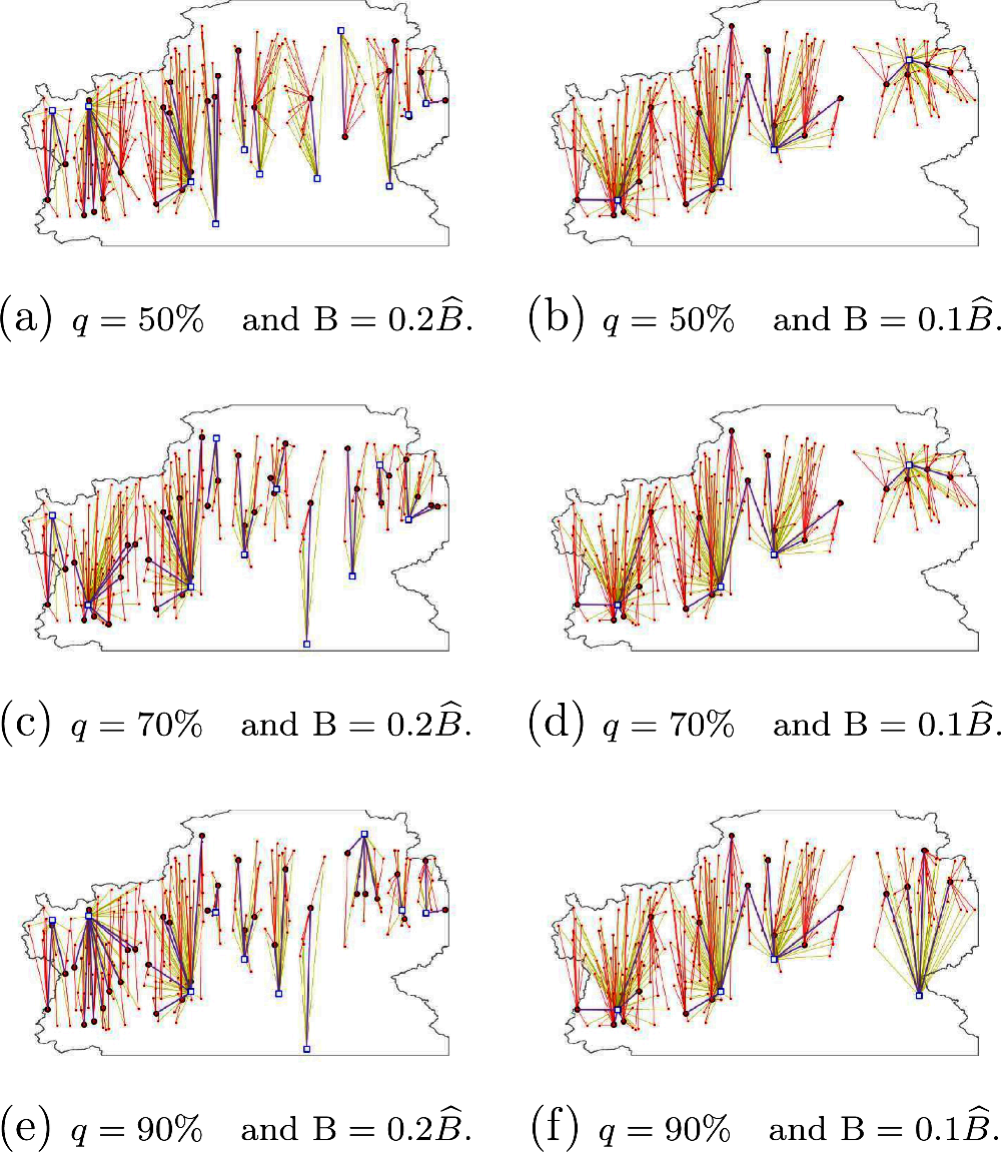
Distribution network between WS, PT plants, and AD plants for the different configurations of *q* and *B*: (a) *q* = 50% and *B* = 0.2*B̂*, (b) *q* = 50% and *B* = 0.1*B̂*, (c) *q* = 70% and *B* = 0.2*B̂*, (d) *q* = 70% and *B* = 0.1*B̂*, (e) *q* = 90% and *B* = 0.2*B̂*, and (f) *q* = 90% and *B* = 0.1*B̂*.

**Figure 8:**
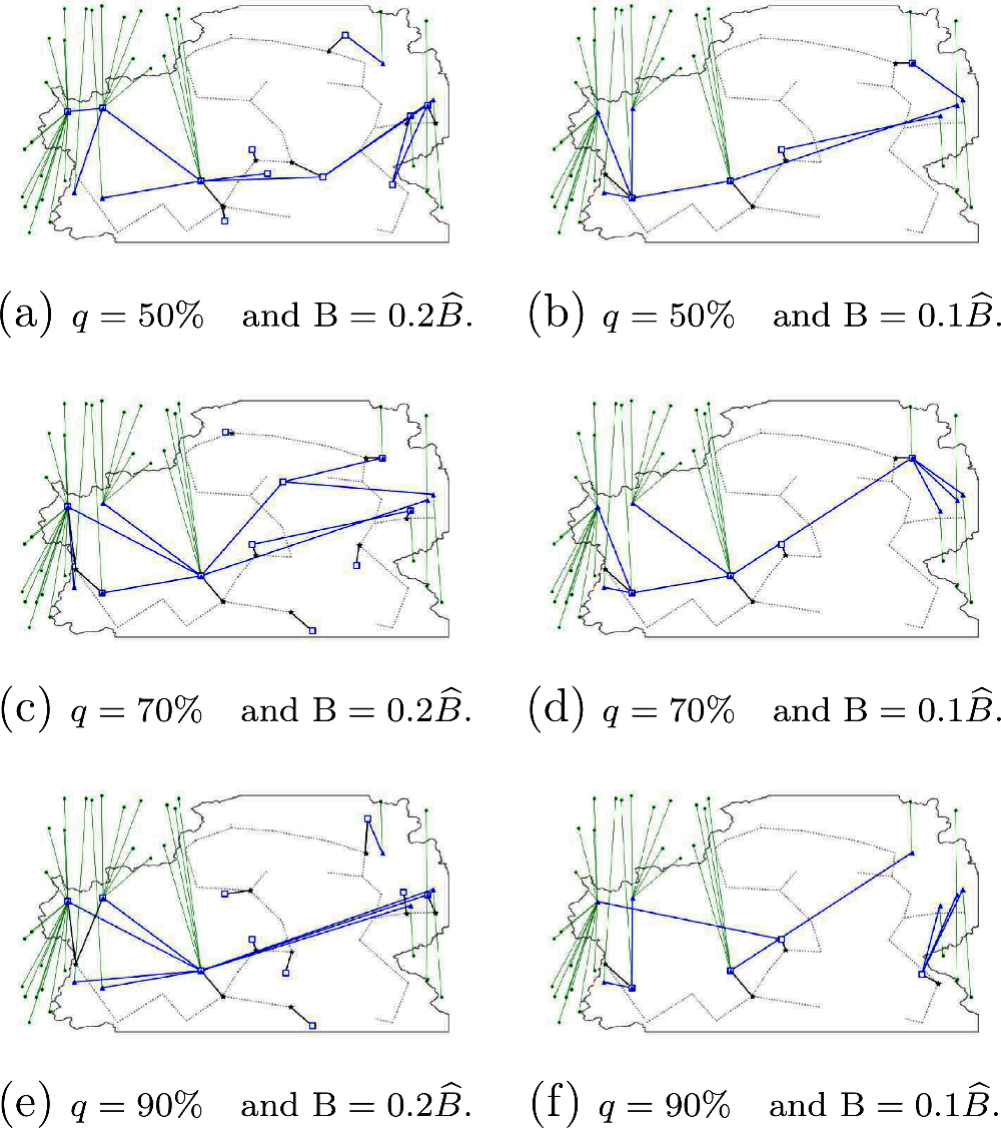
Distribution network between AD plants, BL plants, injection points of the GPN, and EC for the different configurations of *q* and *B*: (a) *q* = 50% and *B* = 0.2*B̂*, (b) *q* = 50% and *B* = 0.1*B̂*, (c) *q* = 70% and *B* = 0.2*B̂*, (d) *q* = 70% and *B* = 0.1*B̂*, (e) *q* = 90% and *B* = 0.2*B̂*, and (f) *q* = 90% and *B* = 0.1*B̂*.

In Figures 6, 7, and 8, we show the results of our experiment for the Yahara watershed dataset for the different choices of budget, *B*, and *q*, and the different decisions that are made by our model.

In Figure 6, we show the results regarding the optimal locations for the PT plants (thick red points), the AD plants (blue squares), and the BL plants (blue triangles), as well as the optimal locations for the links connecting AD plants with BL plants (blue lines) and AD plants with injection points of the GPN (black lines). As can be observed, the budget and the percentage of production that must be injected in the GPN have a direct impact on the structure of the obtained solutions. On the one hand, as expected, the smaller the budget, the smaller the number of plants and pipelines that are open. In particular, the budget mostly affects the construction of pipelines to either inject to the GPN or transport to the BL, but it also affects the number of installed plants. We also observed that the network of the different installed plants and pipelines has more connected components for the larger budget, whereas it is almost connected for the small budget. Regarding the value of *q*, it seems that the larger the value of *q*, the closer the new facilities to the GPN.

In Figure 7, we show the distribution network between the WS plants, PT plants, and AD plants. Specifically, the figure shows the links for which there is a positive waste flow from WS to PT plants (red lines), a DOW flow from PT plants to AD plants (purple lines), and digester solid flow from AD plants to WS (yellow lines). The main observation that can be drawn from these plots is that, in most of the cases, there are differentiated clusters of WS plants (farms) that share the same PT plants and AD plants. This observations implies that, for larger datasets, where the model could not be able to solve the problem, the optimal solution would be adequately approximated by clustering the WS (with an adequate criterion) and solve the problem separately for each cluster.

Finally, in Figure 8, we show the biomethane flow from AD plants to BL plants (blue lines) and to injection points of the GPN (black lines), and LGN flow from BL plants to EC (green lines). Note that some of the AD plants are devoted only to give service to the GPN; others serve the BL plants (and then the EC) exclusively. However, one can also find AD plants that send part of the production to the GPN and the remainder is sent to the BL plants. This behavior would never happen if an integrated model, such as the one that we propose, would not have been considered. Note also that a single AD plant is allowed to send biomethane to different BL plants.

## Conclusions

In this paper, we propose a mathematical optimization approach to design an optimal logistic system to distribute the different products involved in the generation of biogas from waste. Specifically, given a set of waste storage centers, an existing gas pipeline network, and a set of external customers, we provide a decision aid tool to determine the number and optimal locations of pretreatment plants, anaerobic digestion plants, and biomethane liquefaction plants, as well as the pipelines linking some of these plants. Additionally, we provide a distribution plan to send the different wastes, to serve either the gas pipeline network or the external customers with the produced biogas and to serve the waste storage centers with the generated fertilizer. All of the decisions are made by minimizing the transportation costs of the different products and restricting the installation of plants and pipelines to a given budget.

We developed a new mixed integer linear programming (MILP) formulation for the problem and proved some results that allow us to reduce the size of the model. With this simplification, we were able to obtain optimal solutions for this problem in real-world instances.

We report the results of an extensive battery of synthetic computational experiments, and we conclude that our approach is suitable to be applied to different settings and sizes. We also analyze the case study of the Yahara watershed and study the obtained solutions based on different parameters.

Our future research on this topic includes the incorporation of uncertainty in some parameters of the model. Concretely, the production of waste in farms or waste storages is known to vary over the different seasons of the year, and this production may have an impact in the solution of the problem. We will design stochastic optimization models that take to account this uncertainty to construct a robust solution of the W2BLP. Additionally, we will consider capacity constraints for the different plants and multiple periods for the decisions made in our model. Instead of assuming that the plants and pipelines are installed here and now, we decide in which period of time each of the installations is constructed and used, assuming that they have a limited capacity and that a budget is available for each period. The integrated model that considers uncertainty, capacity constraints, and multiperiod decisions will be closer to reality, but the mathematical programming model for it would be prohibitive, even for small instances. Thus, a different solution approach will have to be designed to solve it, which would not be exact but heuristic.
